# Eye, Ear, Nose and Throat Nursing

**Published:** 1905-05

**Authors:** 


Eye, Ear, Nose and Throat Nursing. By A. Edward Davis, A. M., M. D., Professor of Diseases of the Eye in the New York Post-Graduate Medical School and Hospital, and Beaman Douglass, M.D., Professor of Diseases of the Nose and Throat in the New York Post-Graduate Medical School and Hospital. With 32 illustrations. Pages xvi.-318. Philadelphia: F. A. Davis Company. 1905. (Price, $1.25.)

In this work which is intended as a manual for eye, ear, nose, and throat nursing, Davis furnishes the part dealing with the diseases of the eye, and Douglass with that of the ear, nose, and throat. The book contains much valuable information, and is a trustworthy guide to the subjects of which it treats. If there is any criticism at all, it is that which may be applied to most works on nursing—namely, that too much stress and space is given to diseases and to medical and surgical therapeutics, which more properly belong to books for the medical student and physician. Notwithstanding this clinical and therapeutic emphasis the book is to be commended. A. A. H.

